# NGS-based expanded carrier screening for genetic disorders in North Indian population reveals unexpected results – a pilot study

**DOI:** 10.1186/s12881-020-01153-4

**Published:** 2020-11-02

**Authors:** Kanika Singh, Sunita Bijarnia-Mahay, V. L. Ramprasad, Ratna Dua Puri, Sandhya Nair, Sheetal Sharda, Renu Saxena, Sudha Kohli, Samarth Kulshreshtha, Indrani Ganguli, Kanwal Gujral, Ishwar C. Verma

**Affiliations:** 1 Institute of Medical Genetics and Genomics, Sir Ganga Ram Hospital, New Delhi, India; 2 Medgenome Laboratories Pvt Ltd., Bangalore, India; 3 Institute of Obstetrics and Gynaecology, Sir Ganga Ram Hospital, New Delhi, India

**Keywords:** Carrier screening, Cystic fibrosis, Hearing loss, Pompe disease, Asian Indians

## Abstract

**Background:**

To determine the carrier frequency and pathogenic variants of common genetic disorders in the north Indian population by using next generation sequencing (NGS).

**Methods:**

After pre-test counselling, 200 unrelated individuals (including 88 couples) were screened for pathogenic variants in 88 genes by NGS technology. The variants were classified as per American College of Medical Genetics criteria. Pathogenic and likely pathogenic variants were subjected to thorough literature-based curation in addition to the regular filters. Variants of unknown significance were not reported. Individuals were counselled explaining the implications of the results, and cascade screening was advised when necessary.

**Results:**

Of the 200 participants, 52 (26%) were found to be carrier of one or more disorders. Twelve individuals were identified to be carriers for congenital deafness, giving a carrier frequency of one in 17 for one of the four genes tested (*SLC26A4, GJB2, TMPRSS3* and *TMC1* in decreasing order). Nine individuals were observed to be carriers for cystic fibrosis, with a frequency of one in 22. Three individuals were detected to be carriers for Pompe disease (frequency one in 67). None of the 88 couples screened were found to be carriers for the same disorder. The pathogenic variants observed in many disorders (such as deafness, cystic fibrosis, Pompe disease, Canavan disease, primary hyperoxaluria, junctional epidermolysis bullosa, galactosemia, medium chain acyl CoA deficiency etc.) were different from those commonly observed in the West.

**Conclusion:**

A higher carrier frequency for genetic deafness, cystic fibrosis and Pompe disease was unexpected, and contrary to the generally held view about their prevalence in Asian Indians. In spite of the small sample size, this study would suggest that population-based carrier screening panels for India would differ from those in the West, and need to be selected with due care. Testing should comprise the study of all the coding exons with its boundaries in the genes through NGS, as all the variants are not well characterized. Only study of entire coding regions in the genes will detect carriers with adequate efficiency, in order to reduce the burden of genetic disorders in India and other resource poor countries.

**Supplementary Information:**

The online version contains supplementary material available at 10.1186/s12881-020-01153-4.

## Background

Birth defects, defined as abnormalities of structure and function present from birth, are progressively contributing to a greater proportion of fetal, neonatal, infant and childhood mortality in developing countries. This is due to the decline in infectious and nutritional causes due to extensive use of immunizations, control of diarrheal and respiratory infections, and improvements in health care [1]. In the West, 29.8% of early mortality and 29.2% of chronic problems, are due to birth defects [2]. The major difference in the type of disorders observed in developing and developed countries is a higher incidence of autosomal recessive single gene disorders due to consanguinity and endogamous marriages in the former [2, 3]. WHO estimated that globally 206,000 deaths and about 7% of all neonatal deaths are caused by birth defects [4]. In India, the Sample Registration System Survey during 2010–2013 showed the contribution of congenital malformations to childhood mortality, infant mortality and neonatal mortality as 4.4, 4.6 and 4.0% respectively [5]. In the tertiary care hospitals birth defects contributed from 4.2 to 13.4% of perinatal mortality, making congenital malformations and genetic disorders as the third leading cause of neonatal mortality [6]. The burden of genetic disorders in India has been presented in a number of publications and their prevention through screening has been emphasized [3, 7].

Birth defects, including genetic disorders, can cause significant mortality, diminish productivity and quality of life and cause social stigmatization and economic burden especially in resource poor countries. Their prevention is therefore a priority in all countries.

Most countries carry out prevention by screening for infections during pregnancy by serology, chromosomal disorders by biochemical test and structural abnormalities by ultrasonography. However, population prevalence studies have shown that the number of single gene disorders is almost equal to or exceeds chromosomal disorders and congenital malformations combined [3, 7–9]. The cost of prevention through screening for single gene disorders is much less than the cost of treatment. For example, in Cyprus where thalassemia is common, it was shown that the cost of 8 weeks of prevention was equivalent to the cost of 1 week of treatment of the thalassemia population [10]. The Ministry of Health of Israel reported that the life time health care cost for persons with thalassemia vs the cost of national screening program was in a ratio of 4.22 to 1 [11]. The need to reduce the prevalence of genetic disorders in developing countries is greater now as the new treatments of genetic disorders are exorbitantly expensive and out of reach for these families [12, 13]. Moreover most of this expenditure has to be covered by out of pocket expenses by the patients/parents themselves.

Screening for carriers of single gene disorders such as cystic fibrosis and Tay Sach disease has also been shown to be cost effective [14, 15]. Beauchamp et al. examined the clinical impact of a 176-condition expanded carrier screening and demonstrated its cost-effectiveness to reduce the burden of Mendelian disease as compared with minimal screening [16]. Zhanga et al. considered the impact and cost-effectiveness of offering preventive population genomic screening for BRCA1/2, MLH1/MSH2 genes, cystic fibrosis, spinal muscular atrophy and fragile X syndrome to all young adults (18–25 years) in a single-payer health-care system in Australia, and reported that it would be highly cost-effective, but ethical issues need to be considered [17].

The basic objective of carrier screening is to identify carriers and offer them reproductive options from choosing to marry someone who is not a carrier of the same disease (premarital screening) or prenatal diagnosis. In the event that both the husband and wife are carriers of the same disorder, preimplantation genetic diagnosis (after in vitro fertilisation) or prenatal diagnosis (during early stages of a naturally conceived pregnancy) can be carried out [18]. Screening only those families who have a previously affected child is very inefficient, as majority of affected children are born to couples with no previous family history. Similarly screening only those who have an a-priori increased risk of being a carrier based on their personal and family history or who are consanguineously married, or in couples who are opting for sperm or egg donation (Assisted Reproduction Technologies) would still be an inadequate strategy to identify the carriers of genetic disorders. It is best to screen all couples for the genetic disorders common in that population.

World-wide, carrier screening has evolved from an ancestry-based (e.g. in Jewish populations) to pan-ethnic testing, and from single gene disorders, such as cystic fibrosis or α/β-thalassemia, by Sanger sequencing or hematologic techniques, to multiple disorders through Next Generation Sequencing (NGS) [19]. In the West, carrier screening by NGS was initially limited to targeted genotyping because most of the pathogenic variants in the Caucasian population had been characterized and the results were easier to interpret as the subjects were screened for known variants [19]. This approach is not suitable in resource poor countries as most of the pathogenic variants in different genes have not been characterized. However, screening later shifted to NGS of all coding exons of genes to identify carriers more efficiently. This is more suited in India and other resource-poor countries, identifying only the variants that are pathogenic or likely pathogenic and ignoring variants of uncertain significance.

Carrier screening studies for single gene disorders in India, as a service, have chiefly been carried out for β-thalassemia, based on hematologic technique [20]. Isolated studies for p.Phe508del in cystic fibrosis [21] and p.Trp24Ter in *GJB2* related hearing loss [22] and more recently *SMN1* deletion in SMA (Spinal muscular atrophy) [23] have been performed as research studies. The objectives of the present study were to determine the carrier frequency of variants in 88 genes expected to be common in Asian Indians and to identify the pathogenic or likely pathogenic variants.

## Methods

### Subjects

This study was carried out at Sir Ganga Ram Hospital, a tertiary care multispecialty facility, over a period of 22 months from October 2016 through June 2018. Institutional ethical clearance was obtained prior to commencing the study (Ethical clearance number EC/08/ 16/1066). The molecular analysis was performed at Medgenome Laboratories Ltd., Bangalore. Two hundred unrelated individual (*n* = 101 male, *n* = 99 female) between the age of 20–60 years, visiting the Medical Genetics and Obstetrics and/or Gynaecology out-patient clinic for various reasons unrelated to genetic disorders were enrolled, after pre-test counselling. Individuals known to be carriers of any genetic disease, or with history of a chronic medical disorder or familial genetic disorder were excluded from the study. The relevant history and clinical data of each individual was recorded on standard case record proformas (Supplementary file 1).

### Sample size

A sample size of 200 unrelated individuals was planned for enrolment in this pilot study.

### Statistical analysis

Descriptive analysis was done, and outcome reported as proportion of carriers upon total individuals tested (n/200). Confidence interval was calculated by Wilson method [24].

### Selection of gene panel

The selection of genes followed the Wilson and Jungner criteria [25]. Genes selected were those which cause high impact disorders that have significant effect on lifespan or reduce quality of life; or genes with moderate impact that do not reduce lifespan but impact quality of life; or disorders with significant socioeconomic burden for which couples would consider prenatal diagnosis. Limited literature available on the prevalence of various monogenic disorders in India was reviewed. The study by Ankala et al. [26], summarised the prevalence of galactosemia in India 1:10,300, congenital adrenal hyperplasia (CAH) 1:2600, phenylketonuria 1:18,300, and amino acid disorders 1:3600. The prevalence of childhood hearing loss in India was estimated as 1:500 in one study in 2009 [27]. The true prevalence of cystic fibrosis in India is unknown but suspected to be high in a recent review done by Mandal et al. [28], based on the increased citations in recent years. Lazarin et al., [29] also observed a carrier frequency of 1:40 for cystic fibrosis in South Asian population, much higher than expected from data in India. The genetic register maintained about patients evaluated at our centre was analysed. Eighty eight genes [72 autosomal recessive (AR), 7 X-Linked (XL), 9 autosomal dominant (AD/AR) were selected for testing (Supplementary file 2). A smaller number of disorders were aimed at to develop a short but efficient panel that could be offered at a low cost. Two recessive disorders (cystic megalencephaly and calpainopathy) were included as they are common in an ethnic group (Agrawals) in North India [26]. Familial Hypercholesterolemia, though an autosomal dominant disorder was studied as it is life threatening and early treatment can save lives. The study was planned in coherence with American College of Medical Genetics (ACMG) position statement on prenatal/preconception expanded carrier screening [30]. Some common disorders were excluded either because these are not detectable by NGS technology with accuracy or the disorder can be screened easily by haematological tests. These included β-thalassemia, deletions in *SMN1* (survival motor neuron 1) causing SMA (spinal muscular atrophy), FXS (Fragile X syndrome), and DMD (Duchenne muscular dystrophy). Deletion study of CAH was excluded, although sequencing of the gene was performed. Large copy number variations in any of the 88 genes were also not included, in this sequence – based study.

### Pre and post-test counselling

**Fig. 1:**
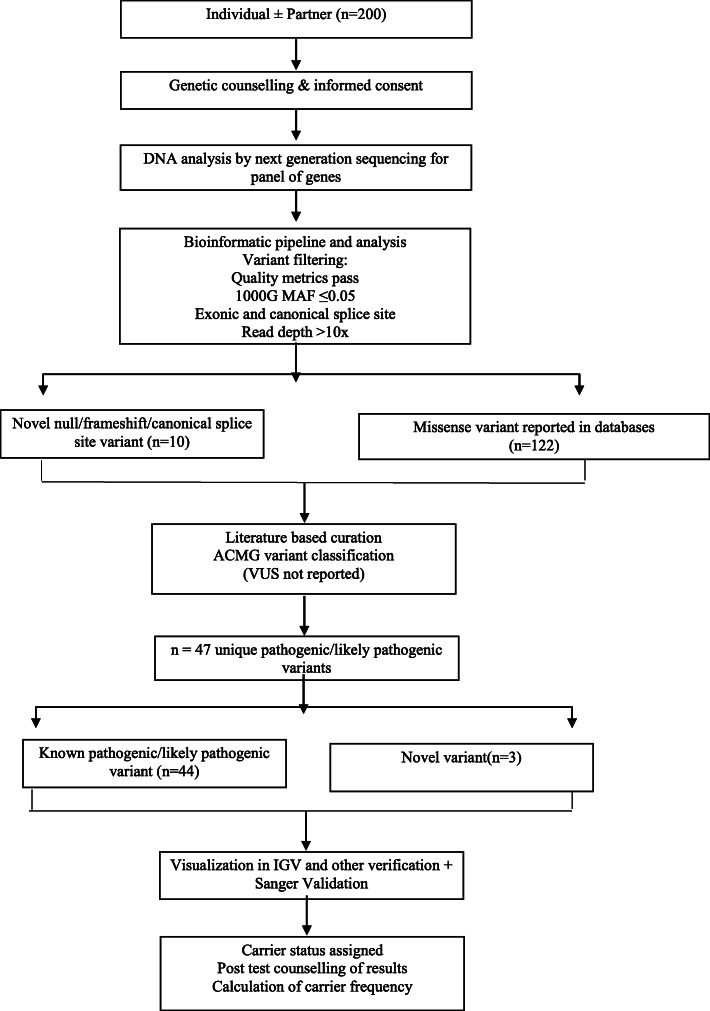
Study flow chart

Prior to the testing, all individuals were counselled about the type of disorders being tested, the implications of being a carrier, the benefits of enrolment of the partner and voluntary nature of testing. Relevant personal, family and ethnic data were recorded. Subjects were clinically examined to rule out any chronic disorder. In post-test counselling the individuals were explained about carrier status and its implications, cascade screening of family members and residual risks remaining after the results (unscreened disorders, chromosomal disorders and indels). The study methodology is depicted in Fig. 1.

### Molecular and Bioinformatic analysis

DNA was extracted from blood using Qiagen kit, and targeted genes were captured by a custom kit. The libraries were sequenced to mean coverage of > 80-100X on Illumina sequencing platform. The sequences obtained were aligned to human reference genome (GRCh37/hg19) using BWA program [31, 32] and analysed using Picard and GATK version 3.6 [33, 34]. Gene annotation of the variants was performed using VEP (Variant effect predictor) program against the Ensembl release 87 human gene model [35]. Clinically relevant pathogenic variants were annotated using published variants in the literature and a set of diseases databases – ClinVar [36], OMIM (Online Mendelian inheritance in man) [37], GWAS catalogue (Genome wide association study in man) [38], HGMD (Human gene pathogenic variant database) [39] and SwissVar [40].

Common variants were filtered based on allele frequency in 1000Genome Phase 3 [41], GnomAD [42], dbSNP147 [43], and an in house database of 100,000 exomes in Indian subjects (Medgenome). Non-synonymous variants effect was calculated using multiple algorithms such as PolyPhen-2 (polymorphism phenotyping v2) [44], SIFT (Sorts intolerant from tolerant) [45], Mutationtaster2 [46], Mutation Assessor [47] and LRT (Likelihood ratio test) [48]. Splicing prediction tools used were Mutationtaster2 [46], BDGP (Berkeley drosophila genome project) [49] and HSF (human splicing finder) [50]. Various filters applied to variants included Variant quality (pass), 1000 genomes MAF (< 0.05), exonic and canonical splice site and read depth (>10x). Variants remaining after applying the listed filters were subjected to ACMG classification. Only those variants fulfilling the ACMG criteria for pathogenic and likely pathogenic were shortlisted [51]. The literature was reviewed for the filtered variants before assigning carrier status.

#### Validation of NGS results

All disease associated variants were manually inspected using IGV (integrative genomics viewer). It was observed that all variants had sequencing depth > 30. No strand biasness was observed. All variants were of good mapping quality. None of the variants were in highly repetitive regions. These were further validated using Sanger sequencing.

## Results

### Population demographics

Of the 200 individuals enrolled, 61.5% belonged to the 31–40 years age group (Table 1). Eighty eight percent had enrolled with their partner and none of them were consanguineously married. Maximum number of persons belonged to the northern states of India (Delhi (*n* = 74), Punjab (*n* = 50), Haryana (*n* = 44), Uttar Pradesh (*n* = 18), Himachal Pradesh (*n* = 5), Jammu and Kashmir (*n* = 5) and Rajasthan (*n* = 4). Majority of the individuals identified themselves as Hindu Punjabi (20.5%). Detailed religious and ethnic characteristics of the subjects is listed in Table 1. Some individuals (5%) could not be classified as they were either unsure of their caste and origin or were born of an inter-caste marriage.

**Table 1:** Demographic characteristics of the individuals enrolled in the study

Parameter			No of individuals (*n* = 200)	Percentage
Age	20–30 yrs		61	30.5%
	31–40 yrs		123	61.5%
	41–50 yrs		13	6.5%
	51–60 yrs		3	1.5%
Sex	Male		101	50.5%
	Female		99	49.5%
Religion & Caste	Hindu	Punjabi	41	20.5%
		Brahmin	30	15%
		Agarwal	28	14%
		Jat	18	9%
		Punjabi	14	7%
		Rajput	6	3%
		Pahadi	6	3%
		BrahminBengali	4	2%
		Marwari	2	1%
		Kashmiri Pandit	2	1%
		Other	10	5%
	Jain		18	9%
	Sikh		12	6%
	Muslim		9	4.5%

### Carrier frequency

Of the 200 participants, 52 (26%) were found to be carrier of one or more disorders (Table 2). Congenital deafness as the most common disorder identified, with a carrier frequency of 1 in 17, for one of the four genes *SLC26A4 (*solute carrier family 26, member 4)*, GJB2 (*gap junction beta 2 protein*), TMPRSS3* (transmembrane protease, serine 3) and *TMC1* (transmembrane channel like protein 1), in decreasing order. Cystic fibrosis was the second most commonly observed disorder with a carrier frequency of 1 in 22. Three subjects were detected to be carriers for Pompe disease (frequency 1 in 67) (Table 2).

**Table 2:** Carrier frequency of the disorders screened

S.no	Disease name (OMIM no.)	N (no. of carriers)/200 individuals	%	1 in _	Wilson 95% Confidence Interval	
					Lower %	Upper %
	Total no. of carrier individuals	52	26	3.84	19.9	31.9
1	Cystic fibrosis - *CFTR* (219700)	9	4.5	22.22	2.4	8.3
2	Deafness - *SCL26A4* (274600)	5	2.5	40.0	0.78	5
3	Deafness - *GJB2* (220290)	3	1.5	66.67	0.5	4.3
4	Deafness - *TMPRSS3* (601072)	3	1.5	66.67	0.5	4.3
5	GSD type II - *GAA* (232300)	3	1.5	66.67	0.5	4.3
6	Methyl malonicaciduria mut A – *MMAA* (251100)	2	1	100	0.27	3.6
7	AR polycystic kidney – *PKHD1* (263200)	2	1	100	0.27	3.6
8	Galactosemia - *GALT* (230400)	2	1	100	0.27	3.6
9	Smith Lemli Opitz syndrome – *DHCR7* (270400)	2	1	100	0.27	3.6
10	Albinism type II - *OCA2* (203200)	2	1	100	0.27	3.6
11	Megalencephalic leukoencephalopathy with cysts -*MLC1* (604004)	2	1	100	0.27	3.6
12	Gaucher disease - *GBA* (230800)	2	1	100	0.27	3.6
13	Phenylketonuria – *PAH* (261600)	2	1	100	0.27	3.6
14	Epidermolysis bullosa (Junctional) -*LAMC2*(226,700, 226,650)	2	1	100	0.27	3.6
15	Niemann Pick disease type C1 – *NPC1* (257220)	1	0.5	200	0.088	2.77
16	Deafness - *TMC1* (600974)	1	0.5	200	0.088	2.77
17	Biotinidase deficiency - *BTD* (253260)	1	0.5	200	0.088	2.77
18	Medium chain acyl CoA deficiency -*ACADM* (201450)	1	0.5	200	0.088	2.77
19	Limb girdle muscle dystrophy type 2A -*CAPN3* (253600)	1	0.5	200	0.088	2.77
20	Congenital adrenal hyperplasia -*CYP21A2*(201910)	1	0.5	200	0.088	2.77
21	Primary hyperoxaluria type 1 - *AGXT* (259900)	1	0.5	200	0.088	2.77
22	Argininosuccinic aciduria - *ASL* (207900)	1	0.5	200	0.088	2.77
23	Canavan disease - *ASPA* (271900)	1	0.5	200	0.088	2.77
24	Glutaric aciduria type 1 – *GCDH* (231670)	1	0.5	200	0.088	2.77
25	Krabbe disease - *GALC* (245200)	1	0.5	200	0.088	2.77
26	Congenital ichthyosis - *TGM1* (242300)	1	0.5	200	0.088	2.77
27	Metachromatic leukodystrophy – *ARSA* (250100)	1	0.5	200	0.088	2.77
28	Zellweger syndrome – *PEX1* (214100)	1	0.5	200	0.088	2.77
29	Epidermolysis bullosa dystrophica – *COL7A1* (226600)	1	0.5	200	0.088	2.77
30	Very long chain acyl CoA dehydrogenase deficiency - *ACADVL* (201475)	1	0.5	200	0.088	2.77

*P* Pathogenic, *LP* Likely pathogenic

There was no couple where both husband and wife were carriers for the same disorder. No woman was found to be a carrier for the seven X-linked disorders included in the panel (Fabry disease, Ornithine transcarbamylase deficiency, hemophilia A and B, Hunter syndrome, severe combined immunodeficiency (SCID) and adrenoleukodystrophy).

Of the 52 (26%) subjects found to be carriers, majority were carriers for one disorder (*n* = 47/200 = 23.5%) and five for two disorders (*n* = 5/200 = 2.5%). No individual was found to be a carrier for three or more disorders.

### Disease causing variants

The disease-causing variants were identified 57 times in 52 of 200 subjects (Table 3). Number of variants were 47, as some variants were identified more than once. Majority were of missense type (72.34%). Among the already reported variants, 29.5% (*n* = 13/44) have been described in patients belonging to the Indian subcontinent (India, Pakistan, Bangladesh). The individual variants are listed in Tables 3 & 4 and discussed in more detail later. Three splice site variants were novel (not reported in the literature or locus specific databases) and fulfilled ACMG criteria for pathogenicity (Table 4).

**Table 3:** Pathogenic and likely pathogenic variants observed in the three commonest disorders

S. no	Disorder and Gene	Transcript no.	Number of Variants	Variant description: cDNA position Protein change	ACMG criteria	No of individuals
1	Deafness, AR 4, with enlarged vestibular aqueduct, *SLC26A4*	ENST00000265715	4	c.1001G > T, p.Gly334Val	PS3 + PM2 + PP2 + PP3 + PP4 + PP5	2
				c.1226G > C, p.Arg409Pro^a^	PM2 + PM5 + PP2 + PP3 + PP5	1
				c.1468A > C, p.Ile490Leu^a^	PS1 + PM1 + PP2 + PP3 + PP5	1
				c.1003 T > C, p.Phe335Leu	PS1 + PP2 + PP3 + PP5	1
2	Deafness, AR,1A, *GJB2*	ENST00000382844	2	c.231G > A, p.Trp77Ter^a^	PVS1 + PS3 + PM1 + PM4 + PP2 + PP3	2
				c.71G > A, p.Trp24Ter^a^	PVS1 + PS3 + PM1 + PM4 + PP2 + PP3	1
3	Deafness, AR, 8, *TMPRSS3*	ENST00000291532	2	c.413C > A, p.Ala138Glu	PM1 + PM2 + PP2 + PP3	1
				c.323-6G > A^a^	PS3 + PS4 + PM2 + PP3	2
4	Deafness AR,7, *TMC1*	ENST00000297784	1	c.1165C > T, p.Arg389Ter	PVS1 + PS3 + PM2 + PM4 + PP3	1
5	Cystic fibrosis, *CFTR*	ENST00000003084	9	c.223C > T, p.Arg75Ter	PVS1 + PS3 + PM2 + PM4 + PP2 + PP3	1
				c.1646G > A, p.Ser549Asn^a^	PS3 + PM1 + PM2 + PM5 + PP2 + PP3 + PP5	1
				c.595C > T, p.His199Tyr	PS3 + PM1 + PM2 + PM5 + PP2 + PP3 + PP5	1
				c.3209G > A, p.Arg1070Gln	PS3 + PM1 + PM5 + PP2 + PP3 + PP5	1
				p.Phe508del	PS3 + PM1 + PM4 + PP2 + PP3	1
				c.4096A > T, p.Ile1366Phe	PM1 + PM2 + PP2 + PP3	1
				c.1472G > T, p.Cys491Phe	PM1 + PM2 + PP2 + PP3	1
				c.4009 T > G, p.Phe1337Val	PM1 + PM2 + PP2 + PP3	1
				c.1859A > T, p.His620Leu	PM1 + PM2 + PM5 + PP2 + PP3	1
6	Glycogen storage disease II, *GAA*	ENST00000302262	1	c.1933G > A, p.Asp645Asn	PS4 + PM1 + PM2 + PM5 + PP2 + PP3 + PP5	3

*PVS* Pathogenic very strong, *PS* Pathogenic strong, *PM* pathogenic moderate, *PP* Pathogenic supporting [51] (Supplementary file 3), ^a^described from the Indian subcontinent

**Table 4:** Pathogenic and likely pathogenic variants in other genes

S. no	Disorder and Gene	Transcript	Number of variants	Variant description: cDNA position Protein change	ACMG criteria	No of individuals
1	Methylmalonic aciduria, cbla type, *MMAA*	ENST00000281317	1	c.433C > T p.Arg145Ter	PVS1 + PS3 + PM4 + PP2 + PP3	2
2	Epidermolysis bullosa dystrophica, AR, *COL7A1*	ENST00000328333	1	c.5287C > T p.Arg1763Ter	PVS1 + PM1 + PM4 + PP2	1
3	Galactosemia, *GALT*	ENST00000378842	1	c.563A > G p.Gln188Arg	PS3 + PM1 + PP2 + PP3 + PP4 + PP5	2
4	Smith-Lemli-Opitz syndrome, *DHCR7*	ENST00000355527	2	c.730G > A p.Gly244Arg	PS1 + PM2 + PP2 + PP3 + PP4 + PP5	1
5				c.862G > A p.Glu288Lys	PM2 + PP1 + PP2 + PP3 + PP4 + PP5	1
6	Biotinidase deficiency, *BTD*	ENST00000303498	1	c.469C > T p.Arg157Cys	PS3 + PM1 + PM2 + PM5 + PP2 + PP3	1
7	Medium chain ACYL-CoA dehydrogenase deficiency, *ACADM*	ENST00000420607	1	c.811G > G/A p.Gly271Arg	PS1 + PS3 + PP2 + PP3 + PP5	1
8	Junctional Epidermolysis bullosa, *LAMB3*	ENST00000391911	1	c.2138-2A > G^a^	PVS1 + PM2 + PP3	2
9	Metachromatic leukodystrophy, *ARSA*	ENST00000216124	1	c.1210 + 1G > T^a^	PVS1 + PM2 + PP3 + PP5	1
10	Peroxisome biogenesis disorder 1A (Zellweger), *PEX1*	ENST00000248633	1	c.2926 + 2 T > C	PVS1 + PM2 + PP3 + PP5	1
11	Polycystic kidney disease, AR, *PKHD1*	ENST00000371117	2	c.8441-1G > C^a^	PVS1 + PM2 + PP3	1
12				c.1480C > T, p.Arg494Ter	PVS1 + PS3 + PM2 + PM4 + PP2	1
13	Albinism type 2, *OCA2*	ENST00000354638	1	c.1580 T > G p.Leu527Arg^b^	PM2 + PP2 + PP3 + PP4 + PP5	2
14	Phenylketonuria, *PAH*	ENST00000553106	1	c.688G > A p.Val230Ile	PS4 + PM1 + PP2 + PP3 + PP5	2
15	Limb Girdle muscle dystrophy type 2A, *CAPN3*	ENST00000397163	1	c.1504A > G p.Ile502Val	PM1 + PM2 + PM5 + PP2 + PP3	1
16	Congenital adrenal hyperplasia, *CYP21A2*	ENST00000418967	1	c.373C > T p.Arg125Cys	PM2 + PP2 + PP3 + PP4 + PP5	1
17	Megalencephalic leukoencephalopathy with subcortical cysts, *MLC1*	ENST00000311597	2	c.65G > A p.Arg22Gln	PS3 + PP2 + PP3 + PP4	1
18				c.959C > A p.Thr320Lys	PS3 + PM2 + PP2 + PP3 + PP5	1
19	Primary hyperoxaluria type 1, *AGXT*	ENST00000307503	1	c.302 T > C p.Leu101Pro^b^	PS3 + PM2 + PP2 + PP3	1
20	Gaucher disease, *GBA*	ENST00000327247	2	c.866G > C p.Gly289Ala^b^	PM2 + PP2 + PP3 + PP4 + PP5	1
21				c.1448 T > C p.Leu483Pro^b^	PS3 + PM5 + PP2 + PP3 + PP5	1
22	Argininosuccinic aciduria, *ASL*	ENST00000304874	1	c.857A > G p.Gln286Arg	PS4 + PP2 + PP3 + PP5	1
23	Canavan disease, *ASPA*	ENST00000263080	1	c.902 T > C p.Leu301Pro^b^	PM2 + PP2 + PP3 + PP4 + PP5	1
24	Glutaric acidemia type 1, *GCDH*	ENST00000222214	1	c.281G > A p.Arg94Gln^b^	PM2 + PM5 + PP2 + PP3 + PP4 + PP5	1
25	Krabbe disease, *GALC*	ENST00000261304	1	c.956A > G p.Tyr319Cys^b^	PM1 + PM2 + PM5 + PP2 + PP3	1
26	Congenital ichthyosis, AR 1, *TGM1*	ENST00000206765	1	c.550C > T p.Pro184Ser	PM2 + PM3 + PP2 + PP3 + PP5	1
27	Very Long chain fatty acid acyl-CoA dehydrogenase deficiency*, ACADVL*	ENST00000543245	1	c.1480 T > C p.Phe494Leu	PM1 + PM2 + PP2 + PP3	1
28	Niemann Pick disease type C1, *NPC1*	ENST00000269228	1	c.3560C > T p.Ala1187Val	PM5 + PP2 + PP3 + PP4 + PP5	1

PVS – pathogenic very strong, PS – pathogenic strong, PM– pathogenic moderate, PP – pathogenic supporting [51] (Supplementary file 3), ^a^Novel variant, ^b^described from the Indian subcontinent

## Discussion

The study was designed to determine the carrier frequency of single gene disorders other than β-thalassemia for which has a carrier frequency of about 3–4% has been shown in many studies in India [52]. Disorders such as spinal muscular atrophy (SMA), fragile X syndrome (FXS) and Duchenne muscular dystrophy (DMD) are common in all populations including South Asians were also excluded as these are difficult to detect with NGS [53]. Recently, we showed the carrier frequency of SMA in North India to be 2.25% [23]. However, carrier frequency for other single gene recessive disorders is not known and significant differences in prevalence and pathogenic variants have been seen in different populations [54].

### *CFTR* (cystic fibrosis transmembrane conductance regulator) pathogenic and likely pathogenic variants

There were nine disease-causing variants identified in the *CFTR* gene in this cohort. Of these, only one case had the common p.Phe508del pathogenic variant i.e. 11% (*n* = 1/9). Two pathogenic variants detected in *CFTR g*ene in this study have been observed before in our laboratory (p.Arg75Ter and p.Ser549Asn). The remaining six pathogenic variants have not been reported in Indians earlier (Table 3). The variants p.Ser549Asn, p.His199Tyr, p.Arg1070Gln have been described by multiple authors and functional studies have been carried out classifying them as pathogenic as per ACMG criteria. The other four variants p.Ile1366Phe, p.Cys491Phe, p.Phe1337Val, p.His620Leu have been documented to be associated with disease, however lack adequate functional studies. They meet the ACMG criteria for likely pathogenicity (Table 3).

*CFTR* c.3854C > T, p.Ala1285Val variant was identified in three individuals, which though has been reported in the literature [55] associated with congenital bilateral absence of vas deferens CBAVD), is more likely to represent a common polymorphism due to its observance in high frequency in the NGS data in the Indian population (0.5% minor allele frequency in South Asians in GnomAD exomes). This variant was not included in the list of disease associated variants.

Studies on the genetic profile of cystic fibrosis patients in India shows high variability, and many rare and new variants have been observed, while only few pathogenic variants (p.Arg1162Ter, p.Met1Thr, c.1161delC, p.Ser549Asp and c.1525-1G > A) are reported more than once [56–58]. This suggests the lack of founder or common mutations in *CFTR* gene and thus emphasises the need for sequencing of all coding regions of the *CFTR* gene in suspected cases in the Indian population. In the present study except for p.Phe508del no other pathogenic variant was present in the ACMG panel of cystic fibrosis [59]. In view of the heterogeneity in pathogenic variants, Mandal et al. also suggested that a single panel of pathogenic variants cannot be used for diagnosis or carrier testing of CF in India [28]. Archibald et al. also observed that the pathogenic variants in cystic fibrosis vary according to ethnic origin [53]. Lim et al. reported in ExAC database that the pathogenic variants in the *CFTR* in non-Europeans are different from those in people of European descent. They noted that none of the current genetic screening panels or existing *CFTR* pathogenic variant databases cover a majority of deleterious variants in any geographical region outside of Europe [60].

Among the nine disease causing variants identified in the *CFTR* gene in the present cohort, only one case had the common p.Phe508del pathogenic variant i.e. 11% (*n* = 1/9). Kapoor and Kabra et al. studied cord blood samples of 955 newborns and reported a p.Phe508del carrier frequency of one in 238 (0.42%) [21]. They estimated the frequency of homozygous p.Phe508del as 1/228,006. However, this cannot be considered to represent the true prevalence of cystic fibrosis in India as it took into account only one pathogenic variant. Comparison of p.Phe508del allele frequency with that reported from the West shows that Indians have a low frequency (19–44%) of the p.Phe508del pathogenic variant [61–63]. Cystic fibrosis was thought to be extremely rare in India. However, a growing number of publications in the last two decades have suggested a higher prevalence [28, 63, 64]. This indicates that CF may be much more common in the Indian population with majority of cases being missed or undiagnosed. *CFTR* related pathogenic variants may be rarely recognized in Indians in view of the different phenotypes (including cystic fibrosis and congenital absence of vas deferens), variable clinical severity and lack of availability of sweat testing, and absence of new born screening.

### *GJB2* c.231G > A, p.Trp77Ter and c.71G > A, p.Trp24Ter

Biallelic variants in the *GJB2* gene or deletion in the gene cause congenital nonprogressive mild to profound sensorineural hearing impairment. The pathogenic variants identified in GJB2 represent have been previously reported in Indian subjects. Ram Shankar et al. studied the pathogenic variants in GJB2 gene in Indian patients with deafness and found p.Trp24Ter to be the most common pathogenic variant India [22]. In addition, they documented two other common pathogenic variants p.Trp77Ter and IVS1 + 1G > A. These differ from the common pathogenic variants identified in the Western (c.35delG) [65] and Japanese (c.235delC) and Korean (p.Val37Ile) populations [66, 67].

### *SLC26A4* related hearing loss

Hearing loss due to *SLC26A4* has been reported as third most common cause of hearing loss in a study in a pan-ethnic population [68]. This occurs due to an enlarged vestibular aqueduct and temporal bone abnormalities which can be appreciated on imaging. In addition to hearing loss, these individuals may have euthyroid goitre (Pendred syndrome). In this study, two out of the four disease - causing variants reported have been previously described in individuals of Indian ethnic origin: p.Arg409Pro [69, 70] and p.Ile490Leu [71]. Other variants found in our study include p.Gly334Val, that has been described chiefly in people of Mediterranean origin [72] and p.Phe335Leu which is a common variant reported worldwide [73].

Carrier screening and prenatal diagnosis for a disorder like hearing loss which impairs quality of life can have differing perceptions among families in different countries. The parental perceptions in Indian culture where resources are scarce towards congenital hearing loss have been pointed out by Nahar et al. previously [74]. While some families are interested in using the information to help in the management, planning and emotional adjustment to the birth of a child with deafness others opt for discontinuing an affected fetus especially if financial resources are scarce.

### *GBA* c.1448 T > C, and c.866G > C, p.Gly289Ala

Biallelic variants in the *GBA* gene causing a deficiency of acid β-glucosidase and cause Gaucher disease, the most common lysosomal storage disorder in the world [75]. The variant p.Gly289Ala and p.Leu483Pro were observed in one individual in the present cohort. Ankleshwari et al. studied 33 Indian patients with Gaucher disease, and identified p.Leu483Pro as the most common pathogenic variant 60.6% (*n* = 20/33). In addition, they reported p.Gly289Ala as a novel pathogenic variant in a patient with type I disease [76]. Homozygosity for the p.Leu483Pro variant is associated with neuronopathic involvement (type III) ranging from mild oculomotor apraxia to more severe involvement as well as lethal cases of collodion skin baby phenotype [77, 78]. The variant most commonly observed in Western population (p.Asn370Ser) and associated with type I Gaucher disease is observed less commonly in India [77, 79].

### *GAA* c.1933G > A, p.Asp645Asn variant

Biallelic pathogenic variants in the *GAA* gene cause deficiency of acid α-glucosidase resulting in Pompe disease. We observed three individuals to be carriers for p.Asp645Asn variant in the GAA gene. This variant was reported for the first time in 1998 by Huie et al. and demonstrated low enzyme activity with this pathogenic variant in vitro and in vivo [80]. Subsequently this pathogenic variant has been reported in patients affected with infantile onset Pompe disease in several studies [81]. This variant lies in exon 14 of the gene, reported to be a hot spot for this gene [81]. However a study done on Indian ethnic patients reported no hot spots for this gene [82].

### *OCA2* c.1580 T > G, p.Leu527Arg variant

Oculocutaneous albinism type II (tyrosinase positive) is caused by biallelic pathogenic variants in the *OCA2* gene. These individuals acquire small amounts of pigment with age and tend to have less severe visual abnormalities. The p.Leu527Arg variant was observed in heterozygous in two individuals in our cohort. It was reported for the first time by Jowerek et al. in a Pakistani family with some pigmentation of hair [83]. They reported that this pathogenic variant lies in highly conserved residue of amino acids in the transmembrane 8 domain of the protein and segregated with affected member.

### *AGXT* c.302 T > C, p.Leu101Pro variant

Primary hyperoxaluria occurs due to deficiency of the liver peroxisomal enzyme alanine:glyoxylate-aminotransferase encoded by the *AGXT* gene. We observed one carrier (belonging to Punjabi community) for p.Leu101Pro variant in our cohort. This variant was reported for the first time by Williams et al. [84], who demonstrated that the mutant gene protein had less than 1% of normal activity in vitro. Subsequently, Chanchlani et al. documented three patients with primary hyperoxaluria type 1 to have the p.Leu101Pro variant in homozygous state [85]. All the three patients belonged to north India or Pakistan. They suggested a possibility of this being a founder pathogenic variant in India although larger studies and haplotype analysis are required.

### *ASPA c*.902 T > C, p.Leu301Pro

The *ASPA* gene encodes for aspartoacylase enzyme, deficiency of which results in Canavan disease. One individual was found to be carrier for the p.Leu301Pro variant. This variant has been reported by our group previously in a patient of Indian ethnicity with classical Canavan disease and raised urine N-acetyl aspartate [86]. On the basis of the reported literature this variant has classified using ACMG criteria as likely pathogenic.

### *ACADM* c.811G > A, p.Gly271Arg

Biallelic pathogenic variants in *ACADM* affect mitochondrial fatty acid β-oxidation due to deficiency of the enzyme medium-chain acyl-coenzyme A dehydrogenase. The p.Gly271Arg is a well reported pathogenic variant in the *ACADM* gene worldwide. It was observed in one individual in this study. The c.985A > G pathogenic variant commonly seen in the West, believed to be a founder pathogenic variant in Caucasians originating from an ancient Germanic tribe was not observed in the present cohort [87].

Disorders like AR polycystic kidney disease, methyl malonic acidemia, galactosemia, Smith-Lemli Opitz syndrome, oculocutaneous albinism type II, cystic megalencephalic leukoencephalopathy, phenylketonuria and junctional epidermolysis bullosa can be expected to be common in the Indian population as at least two cases were detected among the 200 individuals screened.

Other investigators and our group have identified a number of disorders with founder mutations among the Agarwal community [88, 89]. Carriers for only two of these were identified in the current panel of genes - calpainopathy and megalencephalic leukodystrophy with cysts. The mutations detected are not the common ones noted in the Agarwal community. However there were only 28 individuals in the cohort belonging to the Agarwal community and larger studies are indicated to determine their carrier frequency.

## Conclusions

Carrier screening has been widely offered in the preconception period in the West. However, in India it has mostly been offered to those who have a family history or consanguinity. With decreasing costs of NGS panels, carrier screening is being increasingly utilised in recent times in India. It is likely to become the method of choice to decrease the burden of genetic diseases in India, as treatment is not funded by state agencies and family’s financial resources are scarce. This study also brings out the differences in common pathogenic variants between the West and in Asian Indians, an ethnically distinct population. The variant filtration and interpretation strategies in a healthy population are challenging and literature review is essential before assigning pathogenic status to a variant. With the availability of NGS based testing in India and growing amount of literature on Indian pathogenic variants and their representation in databases, the sensitivity of carrier screening is likely to improve. Targeted genotyping panels like the ‘23 pathogenic variant panel’ developed by ACMG for cystic fibrosis are not suitable and will miss many carriers.

This study highlights the importance of an Indian database in improving the classification of variants. It is creditable that many genetic centres are pooling their data to develop such a database. The high carrier frequency of cystic fibrosis, if substantiated in larger population studies, would be sufficient ground to initiate new-born screening in the Indian population. One major limitation of this study is the small sample size, and a larger studies would be justified to serve as a valuable tool for reducing the burden of genetic disorders.

## Supplementary Information

**Additional file 1.** Case record proforma used in the study to record clinical data.

**Additional file 2: Table S1.** table of disorders tested in the study.

**Additional file 3: Tables S2 and S3.** criteria used to assign pathogenicity to variants (adapted from ACMG, 2015 variant classification criteria).

## Data Availability

The datasets generated and analysed during the current study are available in the European Variation Archive (EVA) repository, under the study browser at https://www.ebi.ac.uk/eva. The project number is PRJEB40310. The direct weblink to the data files is https://www.ebi.ac.uk/ena/browser/view/PRJEB40310 The direct weblink to the human reference genome (GRCh37/hg19) dataset used in this study is as follows: ftp://ftp.ensembl.org/pub/grch37/current/fasta/homo_sapiens/dna/ Accession numbers are also listed in Table 3 and Table 4 and their respective datasets can be found in the Ensemble database. The supplementary tables 2 and 3 provide the evidence of pathogenicity, and classification of pathogenic and likely pathogenic variants according to ACMG guidelines, 2015.

## Abbreviations

ACMG: American College of Medical Genetics
AR: Autosomal recessive
AD: Autosomal Dominant
BDGP: Berkeley drosophila genome project
BWA: Burrow wheeler aligner
CAH: Congenital adrenal hyperplasia
CBAVD: Congenital bilateral absence of vas deferens
DMD: Duchenne muscular dystrophy
ECS: Expanded carrier screening
FXS: Fragile X syndrome
GWAS catalogue: Genome wide association study in man
HGMD: Human gene pathogenic variant database
HSF: Human splicing finder
IGV: Integrative genomics viewer
LRT: Likelihood ratio test
NGS: Next generation sequencing
OMIM: Online mendelian inheritance in man
SCID: Severe combined immunodeficiency
SIFT: Sorts intolerant from tolerant
SMA: Spinal muscular atrophy
VEP: Variant effect predictor
VUS: Variants of uncertain significance
XL: X linked
