# Supplementary material for: NGS-based expanded carrier screening for genetic disorders in North Indian population reveals unexpected results – a pilot study
Source: BMC Med Genet. 2020 Nov 2;21:216. doi: 10.1186/s12881-020-01153-4 (PMC7607710; doi:10.1186/s12881-020-01153-4)
Supplement: Supplementary file 1 — Additional file 1. Case record proforma used in the study to record clinical data. [file 12881_2020_1153_MOESM1_ESM.docx]

**CASE RECORD PROFORMA**

Name:

Age/ Sex:

Address:

Contact no:

Date of Enrollment:

History:

Ethnicity/Caste:

Surname of both the parents:

Consanguinity:

History of known genetic disease in self or any chronic illness, vision/hearing complaints (excluding minor refractive errors):

Family history/Pedigree:

Personal history:

*If pregnant: LMP: Gestation:*

Relevant prior Investigation reports if any:

Examination:

1. General Physical Examination
2. Vitals – Pulse rate, Respiratory rate, Blood pressure
3. Neuromuscular examination - Cranial nerves, Muscle tone, power, reflexes, cerebellar signs
4. Cardiovascular system examination – Heart rate, Apex beat, Heart sounds
5. Abdominal examination – Hernial sites, palpation for organomegaly
6. Respiratory system – Breath sounds
