# Supplementary material for: NGS-based expanded carrier screening for genetic disorders in North Indian population reveals unexpected results – a pilot study
Source: BMC Med Genet. 2020 Nov 2;21:216. doi: 10.1186/s12881-020-01153-4 (PMC7607710; doi:10.1186/s12881-020-01153-4)
Supplement: Supplementary file 2 — Additional file 2: Table S1. table of disorders tested in the study. [file 12881_2020_1153_MOESM2_ESM.docx]

**Supplementary table 1: Disorders tested in this study**

| Sno | Disorder | Inherit-ance | Phenotype OMIM no. | Gene name | Gene function |
| --- | --- | --- | --- | --- | --- |
| 1 | Adrenoleukodystrophy | XL | 300100 | ABCD1 | ATP-Binding Cassette Subfamily D (Peroxisomal fatty acid transport) |
| 2 | Argininosuccinic aciduria | AR | 207900 | ASL | Argininosuccinate lyase  (Urea cycle) |
| 3 | Ataxia telengiectasia | AR | 208900 | ATM | DNA Repair gene family |
| 4 | Albinism type I | AR | 203100, | TYR | Tyrosinase (Melanin biosynthesis) |
| 5 | Albinism type II | AR | 203200 | OCA2 | Melanosomal transmembrane protein |
| 6 | Albinism type III | AR | 203290 | TYRP1 | Tyrosinase related protein 1 |
| 7 | Alkaptonuria | AR | 203500 | HGD | Homgentisate 1,2 dioxygenase (Phenylalanine metabolism) |
| 8 | Alpha 1 antitrypsin deficiency | AR | 613490 | SERPINA1 | Serpin Peptidase inhibitor |
| 9 | Biotinidase deficiency | AR | 253260 | BTD | Biotinidase (Biotin metabolism) |
| 10 | Beta Ketothiolase deficiency | AR | 203750 | ACAT1 | Acetyl-CoA Acetyltransferase (Ketone body metabolism) |
| 11 | Congenital disorder of glycosylation type 1A | AR | 212065 | PMM2 | Phosphomannomutase |
| 12 | Canavan disease | AR | 271900 | ASPA | Aspartoacylase |
| 13 | Citrullinemia | AR | 215700 | ASS1 | Argininosuccinate synthetase 1(Urea cycle) |
| 14 | Cystic fibrosis | AR | 219700 | CFTR | Cystic fibrosis transmembrane conductance regulator |
| 15 | Carnitine palmitoyl transferase deficiency type 1 | AR | 255120 | CPT1A | Carnitine Palmitoyltransferase 1 (Urea cycle) |
| 16 | Congenital adrenal hyperplasia | AR | 201910 | CYP21A2 | Cytochrome P450 family enzyme (Adrenal steroidogenesis) |
| 17 | Limb girdle muscle dystrophy AD 4/AR1 | AD/AR | 618129/253600 | CAPN3 | Calpain 3 (Muscle protein) |
| 18 | Deafness AD 3A/AR 1A | AD/AR | 601544/220290 | GJB2 | Gap junction protein |
| 19 | Deafness AR 18A/ Usher syndrome type 1C | AR | 602092/276904 | USH1C | Harmonin (Inner ear hair cell protein) |
| 20 | Deafness AD 36/AR 7 | AD/AR | 606705/600974 | TMC1 | Transmembrane channel-like protein (Cochlear protein) |
| 21 | Deafness AR 8/10 | AR | 601072 | TMPRSS3 | Transmembrane protease serine 3 (Inner ear signalling pathway) |
| 22 | Deafness AR 12 | AR | 601067 | CDH23 | Cadherin23 (Inner ear hair cell protein) |
| 23 | Epidermolysis bullosa dystrophica | AD/AR | 131750/226600 | COL7A1 | Collagen, type VII  (Epithelial protein) |
| 24 | Epidermolysis bullosa junctional | AR | 226700 | LAMC2 | Laminin, Gamma-2 (Epithelial protein) |
| 25 | Epidermolysis bullosa junctional/ Amelogenesis imperfecta | AR/AD | 226650/104530 | LAMB3 | Laminin, Beta-3 (Epithelial protein) |
| 26 | Epidermolysis bullosa junctional/ epithelial recurrent erosion dystrophy | AR/AD | 226730, 122400 | COL17A1 | Collagen, type XVII  (Epithelial protein) |
| 27 | Epidermolysis Bullosa Junctional | AR/AD | 226650/131800 | ITGB4 | Integrin, Beta-4 (Epithelial protein) |
| 28 | Fanconi anemia, complementation group C | AR | 227645 | FANCC | DNA repair gene (Group C) |
| 29 | Fabry disease | XL | 301500 | GLA | Alpha-Galactosidase |
| 30 | Familial hypercholesterolemia | AD, AR | 143890 | LDLR | Low density lipoprotein receptor (Supression of cholesterol synthesis) |
| 31 | Familial hyperinsulinemic hypoglycaemia 1 | AD, AR | 256450 | ABCC8 | ATP Binding Cassette, subfamily C, Member 8 (pancreatic beta cell potassium channels) |
| 32 | Glutaric aciduria type 1 | AR | 231670 | GCDH | Glutaryl CoA-dehydrogenase |
| 33 | Galactosemia | AR | 230400 | GALT | Galactose-1-phosphate uridyl transferase |
| 34 | Gaucher disease | AR | 230800 | GBA | Acid Beta-glucosidase (lysosomal enzyme) |
| 35 | GM1 gangliosidosis | AR | 230500 | GLB1 | Beta-1 galactosidase |
| 36 | GM2 gangliosidosis | AR | 272800/268800 | HEXA, HEXB | Hexosaminidase A/B |
| 37 | Glycogen storage disease type 1A, 1B and 3 | AR | 232200 | G6PC | Glucose-6-phosphatase |
| 38 | Glycogen storage disease type 1B | AR | 232220 | SLC37A4 | Solute carrier family 37, Member 4  (Glucose-6 Phosphate transporter) |
| 39 | Glycogen storage disease type 3 | AR | 232400 | AGL | Amylo 1,6-glucosidase (Glycogen Debrancher enzyme) |
| 40 | Homocystinuria | AR | 236200 | CBS | Cystathionine Beta-synthase |
| 41 | Hereditary fructose intolerance | AR | 229600 | ALDOB | Aldolase B |
| 42 | Heme oxygenase-1 deficiency | AR | 614034 | HMOX1 | Heme oxygenase-1 |
| 43 | Hemophilia A (factor 8 deficiency) | XL | 306700 | F8 | Coagulation factor 8 |
| 44 | Hemophilia B (factor 9 deficiency) | XL | 306900 | F9 | Coagulation factor 9 |
| 45 | Neurodegeneration with brain iron accumulation 1/HARP syndrome | AR | 234200/607236 | PANK2 | Pantothenate kinase 2 |
| 46 | Ichthyosis, congenital, AR 1 | AR | 242300 | TGM1 | Transglutaminase-1 |
| 47 | Ichthyosis, congenital, AR 4A, 4B (harlequin) | AR | 601277/242500 | ABCA12 | ATP Binding Cassette Subfamily A, Member 12 (Keratinocyte protein) |
| 48 | Krabbe disease | AR | 245200 | GALC | Galactosylceramidase (sphingolipid metabolism) |
| 49 | Meckel Gruber syndrome type 3 | AR | 607361 | TMEM67 | Transmembrane protein 67 (Ciliogenesis) |
| 50 | Medium chain acyl CoA dehydrogenase deficiency | AR | 201450 | ACADM | Acyl-CoA dehydrogenase, medium chain (fatty acid oxidation) |
| 51 | Metachromatic leukodystrophy | AR | 250100 | ARSA | Arylsulfatase A (lysosomal enzyme) |
| 52 | Mucopolysaccharidosis type I | AR | 607014 | IDUA | Alpha-L-Iduronidase (lysosomal enzyme) |
| 53 | Mucopolysaccharidosis type II | XL | 309900, | IDS | Iduronate 2-Sulfatase (lysosomal enzyme) |
| 54 | Mucopolysaccharidosis type IVA | AR | 253000 | GALNS | Galactosamine-6-Sulfate Sulfatase (lysosomal enzyme) |
| 55 | Mucopolysaccharidosis type VI | AR | 253200 | ARSB | Arylsulfatase B (lysosomal enzyme) |
| 56 | Maple syrup urine disease type Ia | AR | 248600 | BCKDHA | Branched chain keto acid dehydrogenase E1, Alpha Polypeptide |
| 57 | Maple syrup urine disease type Ib | AR | 248600 | BCKDHB | Branched chain keto acid dehydrogenase E1, Beta Polypeptide |
| 58 | Maple syrup urine disease type II | AR | 248600 | DBT | Dihydrolipoamide branched-chain transacylase |
| 59 | Methyl malonicaciduria mut A | AR | 251100 | MMAA | MMAA (Cobalamin translocation) |
| 60 | Methyl malonicaciduria mut B | AR | 251110 | MMAB | Cobalamin Adenosyltransferase |
| 61 | Methyl malonicaciduria mut C | AR | 277400 | MMACHC | Metabolism of Cobalamin associated C |
| 62 | Megalencephalic leukoencephalopathy with subcortical cysts | AR | 604004 | MLC1 | Modulator of VRAC Current 1 (Astrocyte junctions) |
| 63 | Nonketotic hyperglycinemia | AR | 605899 | GLDC | Glycine decarboxylase |
| 64 | Neuronal ceroid lipofuscinosis type 1 | AR | 256730 | PPT1 | Palmitoyl-Protein Thioesterase 1 (Metabolism of lipid modified proteins) |
| 65 | Neuronal ceroid lipofuscinosis type 2 | AR | 204500 | TPP1 | Tripeptidyl Peptidase 1 (lysosomal exopeptidase) |
| 66 | Neuronal ceroid lipofuscinosis type 3 | AR | 204200 | CLN3 | Lysosomal transmembrane protein |
| 67 | Niemann Pick type A and B | AR | 257200/ 607616 | SMPD1 | Sphingomyelin phosphodiesterase 1 (lysosomal enzyme) |
| 68 | Niemann Pick type C1 | AR | 257220 | NPC1 | Intracellular cholesterol transporter 1 |
| 69 | Niemann Pick type C2 | AR | 607625 | NPC2 | Intracellular cholesterol transporter 2 |
| 70 | Osteopetrosis AR 1 | AR | 259700 | TCIRG1 | T cell immune regulator 1 |
| 71 | Ornithine transcarbamylase deficiency | XL | 311250 | OTC | Ornithine carbamoyltransferase |
| 72 | Phenylketonuria | AR | 261600 | PAH | Phenylalanine hydroxylase |
| 73 | Propionicacidemia | AR | 606054 | PCCA | Propionyl-CoA carboxylase, Alpha subunit |
| 74 | Propionicacidemia | AR | 606054 | PCCB | Propionyl-CoA carboxylase, Beta subunit |
| 75 | Polycystic kidney disease 4 | AR | 263200 | PKHD1 | Ciliary IPT domain containing fibrocystin |
| 76 | Glycogen storage disease type II (Pompe disease) | AR | 232300 | GAA | Glucosidase, Alpha, Acid (lysosomal enzyme) |
| 77 | Pendred syndrome | AR | 274600 | SLC26A4 | Solute carrier family 26, Member 4 (vestibular aqueduct anion transporter) |
| 78 | Primary hyperoxaluria type 1 | AR | 259900 | AGXT | Alanine glyoxylate aminotransferase (peroxisomal enzyme) |
| 79 | Progressive familial intrahepatic cholestasis type 1 | AR | 211600 | ATP8B1 | ATPase, Class I, Type 8B, Member 1 |
| 80 | Progressive familial intrahepatic cholestasis type 2 | AR | 601847 | ABCB11 | ATP binding cassette, subfamily B, member 11 |
| 81 | Progressive familial intrahepatic cholestasis type 3 | AR | 602347 | ABCB4 | ATP binding cassette, subfamily B, member 4 |
| 82 | Smith Lemli Opitz syndrome | AR | 270400 | DHCR7 | 7-dehydrocholesterol reductase |
| 83 | Severe combined immunodeficiency | XL | 300400 | IL2RG | Interleukin 2 receptor, Gamma |
| 84 | Severe combined immunodeficiency | AR | 102700 | ADA | Adenosine deaminase |
| 85 | Tyrosinemia type 1 | AR | 276700 | FAH | Fumaryl acetoacetate hydrolase |
| 86 | Very long chain acyl CoA dehydrogenase deficiency | AR | 201475 | ACADVL | Acyl-CoA Dehydrogenase, Very long chain |
| 87 | Wilson disease | AR | 277900 | ATP7B | ATPase, Copper transporting, Beta polypeptide |
| 88 | Zellweger syndrome | AR | 214100 | PEX1 | Peroxisome biogenesis factor 1 |
