# Supplementary material for: NGS-based expanded carrier screening for genetic disorders in North Indian population reveals unexpected results – a pilot study
Source: BMC Med Genet. 2020 Nov 2;21:216. doi: 10.1186/s12881-020-01153-4 (PMC7607710; doi:10.1186/s12881-020-01153-4)
Supplement: Supplementary file 3 — Additional file 3: Tables S2 and S3. criteria used to assign pathogenicity to variants (adapted from ACMG, 2015 variant classification criteria). [file 12881_2020_1153_MOESM3_ESM.docx]

Supplementary table 2: Evidence of pathogenicity*

| Very strong | PVS1 | Null variant (nonsense,frameshift, canonical ±1 r splice site, initiation codon, single or multiexon deletion) in a gene where LF is a known mechanism of disease |
| --- | --- | --- |
| Strong | PS1 | Same amino acid change as a previously established pathogenic variant regardless of nucleotide change |
|  | PS2 | De novo (both maternity and paternity confirmed) in a patient with the disease and no family history |
|  | PS3 | Well-established in vitro or in vivo functional studies supportive of a damaging effect on the gene or gene product |
|  | PS4 | The prevalence of the variant in affected individuals is significantly increased compared with the prevalence in controls |
| Moderate | PM1 | Located in a mutational hot spot and/or critical and well-established functional domain (e.g., active site of an enzyme) without benign variation |
|  | PM2 | Absent from controls (or at extremely low frequency if recessive) in Exome Sequencing Project, 1000 Genomes Project, or Exome Aggregation Consortium |
|  | PM3 | For recessive disorders, detected in *trans* with a pathogenic variant |
|  | PM4 | Protein length changes as a result of in-frame deletions/insertions in a nonrepeat region or stop-loss variants |
|  | PM5 | Novel missense change at an amino acid residue where a different missense change determined to be pathogenic has been seen before |
|  | PM6 | Assumed de novo, but without confirmation of paternity and maternity |
| Supporting | PP1 | Co-segregation with disease in multiple affected family members in a gene definitively known to cause the disease |
|  | PP2 | Missense variant in a gene that has a low rate of benign missense variation and in which missense variants are a common mechanism of disease |
|  | PP3 | Multiple lines of computational evidence support a deleterious effect on the gene or gene product (conservation, evolutionary, splicing impact, etc.) |
|  | PP4 | Patient’s phenotype or family history is highly specific for a disease with a single genetic etiology |
|  | PP5 | Reputable source recently reports variant as pathogenic, but the evidence is not available to the laboratory to perform an independent evaluation |

*ACMG standards and guidelines on interpretation of sequence variants, 2015 [51]

Supplementary table 3: Classification of pathogenic and likely pathogenic variants*

| Pathogenic | (i) 1 Very strong (PVS1) *AND*  (a) ≥1 Strong (PS1–PS4) *OR*  (b) ≥2 Moderate (PM1–PM6) *OR*  (c) 1 Moderate (PM1–PM6) and 1 supporting (PP1–PP5) *OR*  (d) ≥2 Supporting (PP1–PP5) |
| --- | --- |
|  | (ii) ≥2 Strong (PS1–PS4) *OR* |
|  | (iii) 1 Strong (PS1–PS4) *AND*  (a)≥3 Moderate (PM1–PM6) *OR*  (b)2 Moderate (PM1–PM6) *AND* ≥2 supporting (PP1–PP5) *OR*  (c)1 Moderate (PM1–PM6) *AND* ≥4 supporting (PP1–PP5) |
| Likely pathogenic | (i) 1 Very strong (PVS1) *AND* 1 moderate (PM1–PM6) *OR* |
|  | (ii) 1 Strong (PS1–PS4) *AND* 1–2 moderate (PM1–PM6) *OR* |
|  | (iii) 1 Strong (PS1–PS4) *AND* ≥2 supporting (PP1–PP5) *OR* |
|  | (iv) ≥3 Moderate (PM1–PM6) *OR* |
|  | (v) 2 Moderate (PM1–PM6) *AND* ≥2 supporting (PP1–PP5) *OR* |
|  | (vi) 1 Moderate (PM1–PM6) *AND* ≥4 supporting (PP1–PP5) |

*ACMG standards and guidelines on interpretation of sequence variants, 2015 [51]
